# Nationwide incidence of and risk factors for undergoing incisional glaucoma surgery following infantile cataract surgery

**DOI:** 10.1038/s41598-024-66559-z

**Published:** 2024-07-15

**Authors:** Sooyeon Choe, Young Kook Kim, Ahnul Ha

**Affiliations:** 1 Department of Ophthalmology, Chungnam National University College of Medicine, Daejeon, Korea; 2 Department of Ophthalmology, Chungnam National University Hospital, Daejeon, Korea; 3 Department of Ophthalmology, Seoul National University College of Medicine, Seoul, Korea; 4 Department of Ophthalmology, Seoul National University Hospital, Seoul, Korea; 5 Department of Pediatric Ophthalmology, Seoul National University Children’s Hospital, Seoul, Korea; 6 Clifton Center for Biosocial Informatics, Seoul National University College of Medicine, Seoul, Korea; 7 Department of Ophthalmology, Jeju National University Hospital, Jeju-si, Korea; 8 Department of Ophthalmology, Jeju National University College of Medicine, Jeju-si, Korea

**Keywords:** Infantile cataract, Surgery, Congenital, Glaucoma, Incidence, Risk, Risk factors, Glaucoma, Eye diseases, Lens diseases

## Abstract

Nationwide incidence and risk factors for incisional glaucoma surgery post-infantile cataract (IC) surgery in children remain poorly understood. We conducted a population-based cohort study using the Korean national health claims database to identify IC patients diagnosed before age 1 who had IC surgery among all Korean born between 2008 and 2018 (n = 9,593,003). We estimated the annual occurrence of undergoing incisional glaucoma surgery following IC surgery in the general population aged 0–10. The risk factors for incisional surgery including systemic comorbidities and ophthalmic anomalies were analyzed by multivariable logistic regression. Of 650 patients who had undergone IC surgery with a mean (standard deviation [SD]) follow-up period of 6.2 (3.2) years, 92 (14.2%) were diagnosed with glaucoma following infantile cataract surgery (GFICS). Among them, 21 patients (22.8%) underwent incisional glaucoma surgery after a mean (SD) follow-up duration of 5.4 (2.8) years from the diagnosis of GFICS. Median (InterQuartile Range) age at incisional surgery was 4 (2,6) years old. Twenty of 21 patients (95.2%) underwent incisional glaucoma surgery within 3 years of diagnosis of GFICS. No factors, except younger age at glaucoma diagnosis (*P* = 0.03), were associated with undergoing incisional surgery. These findings can better understand the epidemiologic features and clinical courses of GFICS.

## Introduction

Glaucoma is an important vision-threatening complication after surgery for infantile cataract (IC)^1,2^. The incidence of glaucoma following infantile cataract surgery (GFICS) has been reported to be approximately 15% after a 5-year follow-up period in several studies^3–7^. In one report, secondary glaucoma accounted for up to 58.7% of post-IC-surgery patients.^8^ The primary treatment for GFICS is antiglaucoma eye drops. Although some patients can be managed with medication only, others with refractory glaucoma have to undergo incisional glaucoma surgery^9–11^.

In an effort to avoid vision-threatening post-operative complications after IC surgery, risks for secondary glaucoma have been assessed. Among several possible risk factors for development of GFICS (e.g., young age at IC surgery^7,13,14^, microphthalmia^7,12–15^, and usage of trypan blue^4^), aphakic state after IC surgery^12,16^ was the most controversial, while the Infant Aphakia Treatment Study (IATS) randomized clinical trial (RCT)^17^ identified similar risks of GFICS in both aphakia and pseudophakia groups. Despite previous studies, the incidence of and risk factors for poor outcome (i.e. requiring surgical treatment) of GFICS have not been investigated in population-based data. This may be owed to the low incidence of IC-surgery and the even greater rarity of GFICS.

It was in this context that we recently reported the nationwide birth-cohort incidence of IC surgery and secondary glaucoma risk in South Korean children^16,18^. We have now completed an additional study using a national health claims database obtained through the mandatory universal health insurance system in South Korea. The aim of this study was to report the incidence of and risk factors for undergoing incisional glaucoma surgery following IC surgery in a population-based birth cohort.

## Results

**Figure 1:**
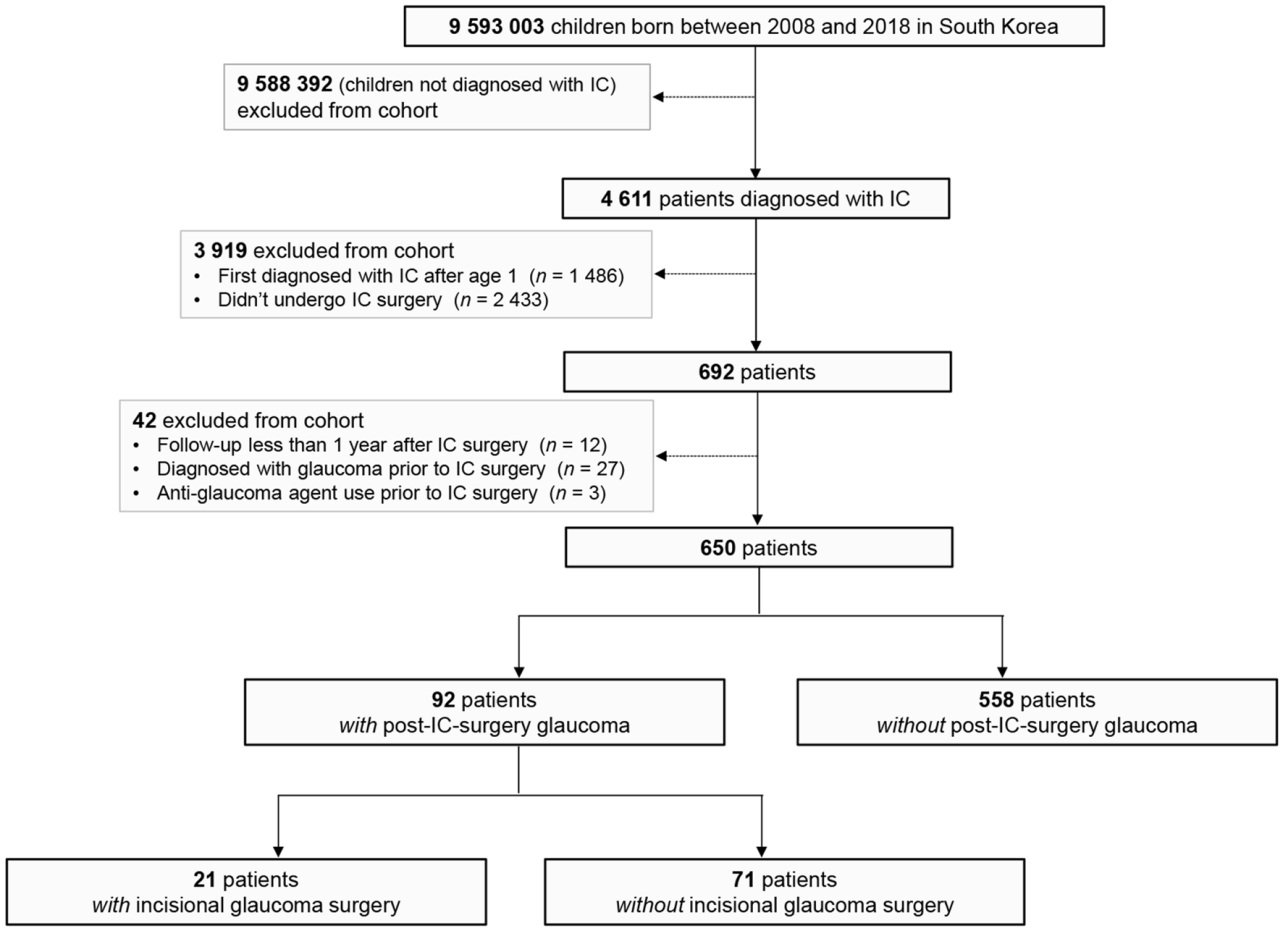
Flowchart of study subjects.

Between 2008 and 2018, 4611 patients were diagnosed with IC. Of 650 IC patients who had undergone IC surgery with a minimum 1-year follow-up period, 92 (14.2%) subsequently were diagnosed with GFICS (Fig. 1). The annual occurrence of GFICS patients is presented in Supplementary Table 1. Among the GFICS patients, 21 patients (22.8%) underwent incisional glaucoma surgery with a follow-up duration of 5.4 ± 2.8 years from the diagnosis of GFICS. Eight (8) of them were male (38.1%), and 17 (81.0%) were bilateral cases. The median (interquartile range [IQR]) ages at diagnosis of GFICS were 3 (1, 5) years among patients with incisional glaucoma surgery and 4 (2, 7) years among those without incisional glaucoma surgery (*P* = 0.053) (Table 1). There were no significant differences in genetic, metabolic, infectious comorbidities or ophthalmic anomalies between the groups with/without incisional glaucoma surgery (Supplementary Table 2).

**Table 1:** Baseline clinical characteristics of GFICS patients with/without incisional glaucoma surgery.

Characteristics	GFICS with incisional surgery (N = 21)	GFICS without incisional surgery (N = 71)	*P* value
Sex, n (%)			0.217^a^
Male	8 (38.1)	39 (54.9)	
Female	13 (61.9)	32 (45.1)	
IC			
Age at diagnosis			0.035^b^
0 < ≤ 28 days	1 (4.8)	20 (28.2)	
28 < ≤ 1 year	20 (95.3)	51 (71.8)	
Age at IC surgery			0.191^b^
0 < ≤ 28 days	0 (0.0)	8 (11.3)	
28 < ≤ 1 year	21 (100.0)	63 (88.7)	
Laterality of IC, n (%)			1.000^b^
Unilateral	4 (19.1)	14 (19.7)	
Bilateral	17 (81.0)	57 (80.3)	
Type of IC surgery, n (%)			0.476^a^
Aphakia	11 (52.4)	28 (39.4)	
Primary IOL	1 (4.8)	8 (11.3)	
Secondary IOL	9 (42.9)	35 (49.3)	
Institution of IC surgery			1.000^b^
City	21 (100.0)	70 (98.6)	
Rural	0 (0.0)	1 (1.4)	
Time from IC diagnosis to IC surgery (days)	19.2 ± 23.4 [12 (0, 28)]	14.2 ± 35.5 [6 (0,18)]	0.204^c^
GFICS			
Observation duration from GFICS to end of study (years)	5.4 ± 2.8 [4.2 (3.3, 7.7)]	2.5 ± 2.3 [1.8 (0.9, 3.2)]	< 0.001^c^
Age at onset of glaucoma (years), median (IQR)	3 (1,5)	4 (2,7)	0.053^c^
Number of types of drugs for glaucoma			0.004^a^
1	1 (4.8)	5 (7.0)	
2	2 (9.5)	35 (49.3)	
3	12 (57.1)	25 (35.2)	
4	6 (28.6)	6 (8.5)	

^a^*P* values were calculated using chi-squared test.

^b^*P* values were calculated using Fisher’s exact test.

^c^*P* values were calculated using Wilcoxon rank sum test.

GFICS glaucoma following infantile cataract surgery, IC infantile cataract, IOL intraocular lens, IQR interquartile range.

### Cumulative incidence of incisional *glaucoma* surgery following IC surgery

**Figure 2:**
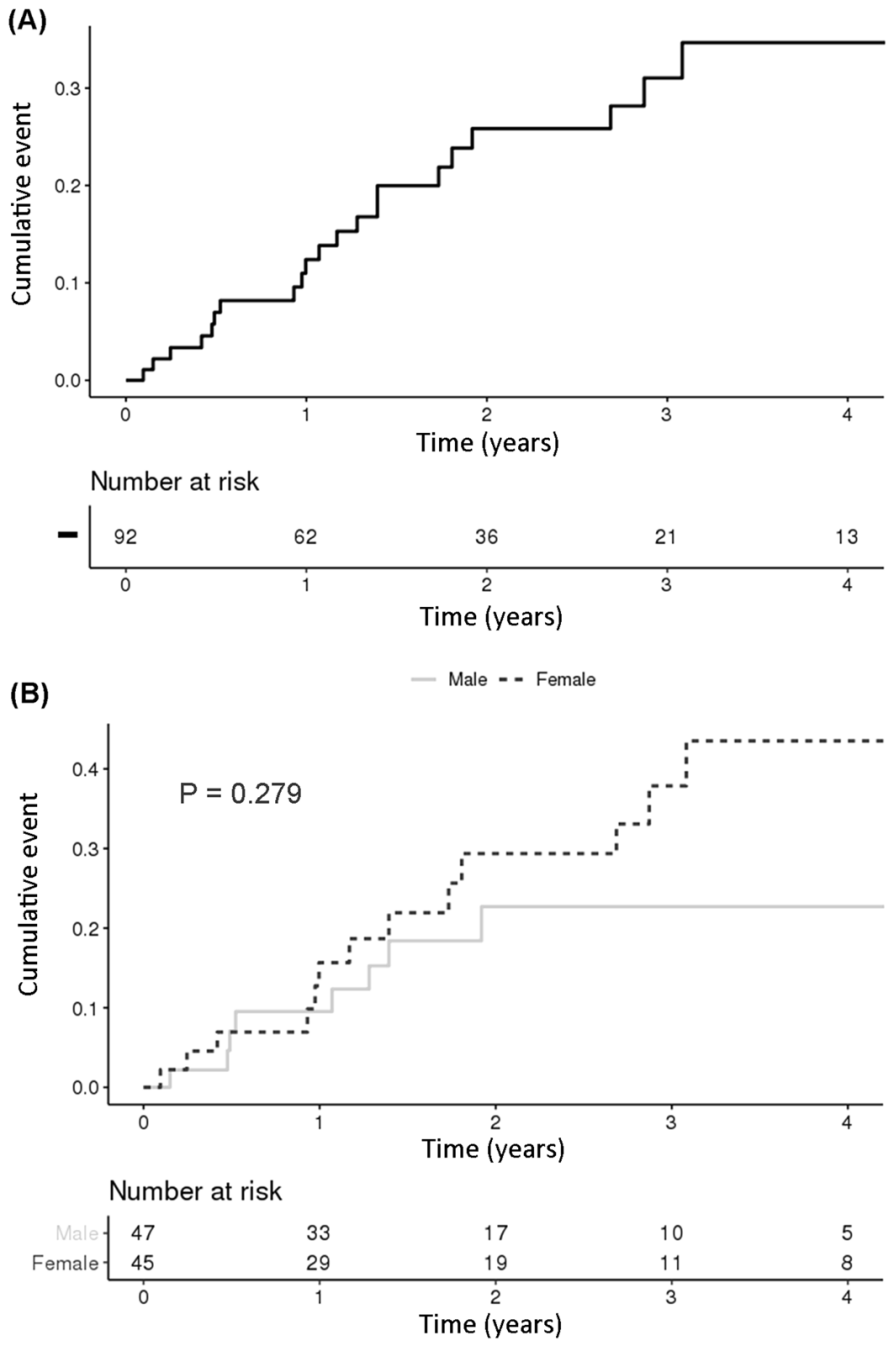
Cumulative incidence graph of GFICS cases with incisional glaucoma surgery. (**A**) Cumulative incidence graph of total population. The incidence of incisional glaucoma surgery gradually increased until 3 years after diagnosis of GFICS. (**B**) Cumulative incidence graph by sex. There was no significant difference between males and females (*P* = 0.279).

The annual occurrence of incisional glaucoma surgery following IC surgery among the general population aged 0–10 is shown in Table 2. The annual occurrence of the patients with incisional glaucoma surgery ranged from 0 to 6 patients. As shown in Fig. 2A, the cumulative incidence increased gradually until 3 years after the GFICS diagnosis. About a quarter of patients underwent incisional glaucoma surgery 0.5 years after diagnosis of GFICS, nearly half underwent incisional glaucoma surgery after 1.1 years, and the remaining quarter, after 1.8 years. There was no statistical difference of cumulative incidence of glaucoma surgery by sex (*P* = 0.279; Fig. 2B).

**Table 2:** Annual occurrence of GFICS patients with incisional glaucoma surgery among general population aged 0–10.

	Total		Male		Female	
	Total Population	Number of cases	Total population	Number of cases	Total population	Number of cases
2009	5,640,054	1	2,930,199	1	2,709,855	0
2010	5,467,961	1	2,836,707	1	2,631,254	0
2011	5,312,764	1	2,751,759	1	2,561,005	0
2012	5,196,539	3	2,686,901	1	2,509,639	2
2013	5,137,703	2	2,652,144	0	2,485,559	2
2014	5,088,822	0	2,622,749	0	2,466,073	0
2015	5,048,970	0	2,599,027	0	2,449,943	0
2016	5,023,852	2	2,583,268	2	2,440,585	0
2017	4,970,318	3	2,553,681	1	2,416,637	2
2018	4,848,344	6	2,489,885	1	2,358,459	5
2019	4,690,395	2	2,407,963	0	2,282,433	2

*GFICS* glaucoma following infantile cataract surgery.

### Characteristics of GFICS patients who underwent *glaucoma* surgery

Of the 21 GFICS patients who underwent incisional glaucoma surgery, the median (IQR) age at incisional glaucoma surgery was 4 (2, 6) years (Table 3). Among them, 10 patients underwent trabeculectomy (TLE), 10 underwent glaucoma drainage implant (GDI) surgery, and one underwent both TLE and GDI surgery. The mean (standard deviation) time from diagnosis of GFICS to glaucoma surgery was 1.2 (0.2) years. Seventeen (17) patients (81.0%) underwent unilateral glaucoma surgery while 4 (19.1%) underwent bilateral glaucoma surgery. Nine patients (9) 42.9%) required reoperation (1 additional surgery for 5 patients; 2 additional surgeries for 2 patients; and 3 or more additional surgeries for 2 patients).

**Table 3:** Clinical characteristics of GFICS patients who underwent incisional glaucoma surgery.

Characteristics	Patients with incisional glaucoma surgery (N = 21)
Age at glaucoma surgery (years), median (IQR)	4 (2, 6)
Age at glaucoma surgery (years) categories, n (%)	
28 < ≤ 1 year	4 (19.1)
2	4 (19.1)
3+	13 (61.9)
Type of glaucoma surgery, n (%)	
Trabeculectomy	10 (47.6)
Glaucoma drainage implant surgery	10 (47.6)
Trabeculectomy + Glaucoma drainage implant surgery	1 (4.8)
Laterality of glaucoma surgery, n (%)	
Unilateral	17 (81.0)
Bilateral	4 (19.1)
Reoperation glaucoma surgery, n (%)	9 (42.9)
Time from GFICS diagnosis to glaucoma surgery (years)	
mean ± SD, [median (IQR)]	1.2 ± 0.2 [1.1 (0.5, 1.8)]
Time from IC surgery to glaucoma surgery (years)	
mean ± SD, [median (IQR)]	4.5 ± 3.3 [3.9 (2.1, 6.7)]
Institution of glaucoma surgery	
Location	
City	21 (100.0)

*GFICS* glaucoma following infantile cataract surgery, *IC* infantile cataract, *IQR* interquartile range, *SD* standard deviation.

### Factors associated with undergoing incisional *glaucoma* surgery following IC surgery

In the multivariable analysis, genetic, metabolic, infectious comorbidities and ophthalmic anomalies were not associated with risk of undergoing incisional glaucoma surgery (Table 4). Likewise, factors associated with IC (i.e., age at IC diagnosis, age at IC surgery, type of IC surgery, and location of IC surgery institution) also were not associated with risk of incisional glaucoma surgery. However, younger age at diagnosis of GFICS showed a higher risk of subsequent incisional glaucoma surgery (*P* = 0.03).

**Table 4:** Multivariable analysis of risk of undergoing incisional glaucoma surgery in GFICS patients.

Characteristics	Univariable		Multivariable	
	OR (95% CI)	*P* value	OR (95% CI)	*P* value
Sex				
Male	1		1	
Female	1.98 (0.73–5.37)	0.18	1.99 (0.65–6.05)	0.23
IC				
Age at diagnosis				
0 < ≤ 28 days	1		1	
28 < ≤ 1 year	7.84 (0.99–62.38)	0.05	4.93 (0.96–31.81)	0.09
Age at IC surgery				
0 < ≤ 28 days	1		1	
28 < ≤ 1 year	5.75 (0.26–123.19)	0.26	1.07 (0.04–32.31)	0.97
Type of IC surgery, n (%)				
Aphakia	1		1	
Primary IOL implantation	0.32 (0.04–2.85)	0.31	0.37 (0.04–3.12)	0.36
Secondary IOL implantation	0.66 (0.24–1.80)	0.41	1.12 (0.30–4.18)	0.86
Institution of IC surgery				
Location				
City	1		1	
Rural	1.12 (0.01–103.38)	0.96	0.50 (0.00–55.37)	0.77
GFICS				
Age at onset of glaucoma	0.13 (0.02–1.01)	0.05	0.80 (0.65–0.99)	0.03
Comorbidities				
None	1		1	
Genetic & metabolic	1.51 (0.17–13.72)	0.71	1.49 (0.16–13.55)	0.73
Infections	2.20 (0.07–69.74)	0.66	2.26 (0.06–79.71)	0.65
Ophthalmic anomalies	0.98 (0.24–3.96)	0.98	1.34 (0.27–6.66)	0.72

*GFICS* glaucoma following infantile cataract surgery, *IC* infantile cataract, *OR* odds ratio, *CI* confidence interval, *IOL* intraocular lens.

## Discussion

Our study was a nationwide, population-based investigation of the incidence of and factors associated with poor outcome of GFICS. Twenty-one (21) patients (22.8%) underwent incisional glaucoma surgery after a follow-up duration of 5.4 years from the diagnosis of GFICS. Twenty (20) of 21 patients (95.2%) underwent incisional glaucoma surgery within 3 years of diagnosis of glaucoma. Younger age at glaucoma diagnosis was associated with higher risk of glaucoma surgery.

To our knowledge, this is the first report of a nationwide incidence and risk of refractory GFICS in East Asia. In previous single-center studies on Western populations, Bhola et al.^19^ reported that 27% of aphakic glaucoma required glaucoma surgery, and Chen et al.^1^ demonstrated that 57.1% needed glaucoma surgery. In the 10-year IATS study^17^, 11 of 25 eyes (44%) underwent glaucoma surgery, while in another RCT by Vasavada et al.^5^, 8 of 54 eyes (14.8%) underwent glaucoma surgery. These widely varying occurrence rates of refractory glaucoma may have been owed to their small number of cases, along with differences of ethnicities and study designs. Our database, which constitutes a nationwide cohort, can present a more representative occurrence rate (22.8%) of surgery for GFICS in a general population.

In the current study, older age at glaucoma diagnosis showed a lower OR for incisional glaucoma surgery, indicating that young age at glaucoma diagnosis was a poor prognostic factor for GFICS patients. There have been few studies analyzing risk factors for glaucoma surgery in GFICS patients. Interestingly, although young age at IC surgery^5–7,20–22^ and type of cataract surgery^16,23–25^ are considered to be risk factors for development of glaucoma after IC surgery, they were not associated with poor outcome of GFICS in our results. Patients with early-onset glaucoma after IC surgery may reflect a higher risk of glaucoma surgery; thus, monitoring with extra caution are warranted. Notably, GFICS patients who had undergone incisional surgery had a significantly longer observation duration from GFICS to the end of the study compared with those without incisional surgery. This may be attributed to the fact that patients who undergo incisional surgery tend to be diagnosed with glaucoma at a younger age. It is well known that the cumulative risk of secondary glaucoma following IC surgery increases with patient age^16,17^. The cumulative risk and incidence of glaucoma surgery following IC surgery would escalate with longer follow-up duration and increasing patient age, possibly altering the results observed in our analysis.

With regard to the method of surgical management, TLE and GDI implantation were performed in the same proportions among GFICS patients between 2009 and 2019 in South Korea. Since the Ahmed glaucoma valve (AGV) was the only glaucoma drainage implant accessible in South Korea throughout the study period, GDI implantation can be referred to as AGV implantation. Pakravan et al.^11^ showed in an RCT that both TLE and Ahmed glaucoma valve (AGV) implantation had comparable success and complication rates. Geyer et al.^26^ recently reported that AGV implantation was successful, having shown an 83.1% success rate at 5 years. Although AGV implantation may be expected to have a relatively high success rate, tube-related complications, such as infection, tube exposure, or corneal decompensation must be given serious consideration, especially for pediatric patients. For the purposes of finding a consensus on a procedure of choice for refractory GFICS, long-term follow-up studies with larger sample sizes will be necessary.

Our study has several limitations. First, the Health Insurance Review and Assessment (HIRA) database did not include clinical information useful for understanding of refractory GFICS. Since the HIRA database consists of medical claims data and does not afford access to the medical records of each patient, some variables such as visual acuity, intraocular pressure, and gonioscopic appearance were not obtainable. Further study including these clinical factors may demonstrate additional risk factors associated with refractory GFICS. Second, since clinical data on abnormalities of optic disc or cornea were not accessible, a possibility of over- or under-estimation of GFICS should be taken into account. To mitigate this limitation, we included only those who had the glaucoma diagnosis code (H40) and a glaucoma treatment history. Third, this study is retrospective in nature. While prospective studies do enable access to high-level evidence, they are infeasible in terms of the need to answer research questions regarding cases of rare diseases. To minimize the present study’s methodological bias arising from its retrospective design, we registered a predefined study protocol. Fourth, given that the HIRA database’s healthcare data is for people residing in and receiving treatment in South Korea, some patients undergoing IC surgery outside of Korea would not have been included therein. Fifth, as the HIRA database contains claims data, instances of GFICS mistakenly classified under other glaucoma diagnostic codes such as congenital glaucoma (Q150) could possibility have been left out.

In conclusion, the present study determined the occurrence rate and risk factors for refractory GFICS in a nationwide cohort. After a follow-up duration of 5.4 years from the diagnosis of GFICS, 21 patients (22.8%) underwent incisional glaucoma surgery. Among them, 20 patients (95.2%) underwent incisional glaucoma surgery within 3 years of diagnosis of glaucoma. Younger onset of GFICS was associated with higher risk of refractory glaucoma. These findings can be of help to management of GFICS patients in clinical settings.

## Methods

This project was approved by the Institutional Review Board (IRB) of Seoul National University Hospital, Seoul, South Korea (IRB No. E-2011–103-1173), and was conducted in accordance with the Declaration of Helsinki. Because this study analyzed anonymized and de-identified data, the requirement for informed consent was waived by the IRB of Seoul National University Hospital, Seoul, South Korea (IRB No. E-2011–103-1173).

### Data

The methodologic details of this study, including case definitions, data sources and data collection, have been reported previously^16,18^. As part of the Korean Nationwide Epidemiological Study for Childhood Glaucoma (KoNEC), which plans for an epidemiological investigation of both primary glaucoma and secondary glaucoma^16,18,27,28^ in Korean children, the protocol was preregistered in the Open Science Framework (OSF; registration number, 10.17605/OSF.IO/AWTEC).

For our retrospective cohort investigation, we accessed the HIRA database between 2008 and 2019. This database includes demographic information as well as information related to the medical records and expenses of enrollees, such as diagnoses, dates of medical visits, examinations, prescriptions, and procedures. All full-time admissions, outpatient visits, and hospital emergency department contacts with a hospital were screened. Diagnoses were recorded according to the international classification of diseases, 10th Revision (ICD-10).

The HIRA deliberative committee approved the conditional use of the HIRA database for the purposes of this study, based on the understanding that all identifiable personal information would be de-identified and the Korean resident registration number would be replaced with a randomly assigned identification key for each patient.

### Participants

All children born in South Korea from 2008 through 2018 (n = 9 593 003) were included. Since our study primarily aimed to ascertain the incidence of glaucoma surgery post IC surgery and identify associated risk factors, we excluded patients with probable pre-existing glaucoma. In detail, we excluded (1) those with a diagnosis of glaucoma prior to IC surgery, and (2) those prescribed an antiglaucoma agent prior to IC surgery. Cases of IC with concomitant non-ocular and ocular anomalies were included for analysis, with their associated factors examined. Non-ocular anomalies encompass genetic, metabolic, and infectious comorbidities known to be associated with IC^29^ (Supplementary Table 2).

### Exposure

We established a cohort of children who had undergone IC surgery between January 2008 and December 2018. Among them, patients who were diagnosed as secondary glaucoma after IC surgery were identified. GFICS was defined as carrying the glaucoma diagnosis code (H40) and having been treated with one or more antiglaucoma agents for longer than 3 months or having a history of glaucoma surgery. For bilateral cases, we selected the eye that had first exhibited glaucoma. If the diagnosis was concurrent in both eyes, the right eye was preferentially chosen.

### Outcome

The primary outcome was incidence of incisional glaucoma surgery following IC surgery. Incisional glaucoma surgeries were identified as TLE (S5033, S5043) or GDI surgery (S5049). Accordingly, the GFICS cases were classified as (1) patients *with incisional glaucoma surgery* and (2) those *without incisional glaucoma surgery*.

### Follow-up

Participants were observed between January 1, 2008 and December 31, 2019. People migrating out of the country or who had died were censored on the relevant date.

### Statistical analysis

The annual occurrence of GFICS patients undergoing incisional glaucoma surgery was estimated as the number of patients undergoing surgery for secondary glaucoma in the general population aged 0–10. The cumulative incidence of GFICS cases with incisional glaucoma surgery was estimated as the number of patients undergoing incisional glaucoma surgery among GFICS patients. Kaplan–Meier survival analysis was used to draw the cumulative incidence curve of incisional glaucoma surgery, and log-rank testing was performed to compare the curves by sex. We incorporated a comprehensive set of adjustment variables known to be possibly associated with risk of GFICS. A multivariable analysis of the risk factors for undergoing incisional surgery among GFICS patients was performed by logistic regression. We included all variables from the univariable analysis to multivariable analysis. All of the analyses were performed with SAS Enterprise Guide version 7.1 (SAS Inc., Cary, NC, USA). Except where otherwise stated, the data are presented as mean ± standard deviations, and the level of statistical significance was set at 2-sided *P* < 0.05.

### Supplementary Information

Supplementary Tables.

## Data Availability

The data that support the findings of this study are available from the HIRA but restrictions apply to the availability of these data, which were used under license for the current study, and so are not publicly available. Data are however available at https://opendata.hira.or.kr/ with permission of the HIRA.

## Abbreviations

*AGV*: Ahmed glaucoma valve
*GDI*: Glaucoma drainage implant
*GFICS*: Glaucoma following infantile cataract surgery
*HIRA*: Health insurance review and assessment
*IATS*: Infant aphakia treatment study
*IC*: Infantile cataract
*IQR*: Interquartile range
*IRB*: Institutional review board
*RCT*: Randomized clinical trial
