# Supplementary material for: Nationwide incidence of and risk factors for undergoing incisional glaucoma surgery following infantile cataract surgery
Source: Sci Rep. 2024 Jul 15;14:16286. doi: 10.1038/s41598-024-66559-z (PMC11251266; doi:10.1038/s41598-024-66559-z)
Supplement: Supplementary file 1 — Supplementary Tables. [file 41598_2024_66559_MOESM1_ESM.pdf]

Supplementary Information

Supplementary Table S1. Annual Occurrence of Post-IC-Surgery Glaucoma Among the General Population Aged 0-11

Supplementary Table S2. Baseline Systemic and Ocular Comorbidities of Post-IC-Surgery Glaucoma Patients with/without Incisional Glaucoma Surgery

Supplementary Table S1. Annual Occurrence of Post-IC-Surgery Glaucoma Among the General Population Aged 0-11[a]

	Total		Male		Female	
	Total population	Number of cases	Total population	Number of cases	Total population	Number of cases
2009	6,296,319	4	3,272,297	3	3,024,022	1
2010	6,097,806	0	3,166,034	0	2,931,772	0
2011	5,942,014	3	3,080,615	1	2,861,399	2
2012	5,795,480	7	2,999,557	4	2,795,923	3
2013	5,664,411	2	2,926,880	0	2,737,532	2
2014	5,582,583	4	2,879,982	0	2,702,601	4
2015	5,532,281	3	2,849,882	2	2,682,399	1
2016	5,478,528	21	2,818,903	9	2,659,625	12
2017	5,412,841	10	2,782,695	7	2,630,146	3
2018	5,320,645	18	2,733,386	10	2,587,259	8
2019	5,172,224	15	2,655,769	8	2,516,456	7

[a] The total population for post-IC-surgery glaucoma was the general population aged 0-11, considering a minimum 1-year follow-up period.

Supplementary Table S2. Baseline Clinical Characteristics of Post-IC-Surgery Glaucoma Patients with/without Incisional Glaucoma Surgery

**Comorbidities, n (%)**	**Post-IC-Surgery Glaucoma *with* Incisional Surgery (N=21)**	**Post-IC-Surgery Glaucoma *without* Incisional Surgery (N=71)**	***P* value**
Genetic & Metabolic	1 (4.8)	5 (23.8)	1[a]
Down syndrome	0 (0.0)	0 (0.0)	
Hallermann-Streiff syndrome	0 (0.0)	0 (0.0)	
Lowe syndrome	1 (4.8)	4 (5.6)	1[a]
Galactosemia	0 (0.0)	1 (1.4)	1[a]
Marfan syndrome	0 (0.0)	0 (0.0)	
Trisomy 13-15	0 (0.0)	0 (0.0)	
Neonatal hypoglycemia	0 (0.0)	0 (0.0)	
Alport syndrome	0 (0.0)	0 (0.0)	
Myotonic dystrophy	0 (0.0)	0 (0.0)	
Fabry disease	0 (0.0)	0 (0.0)	
Hypoparathyroidism	0 (0.0)	0 (0.0)	
Conradi-hunermann syndrome	0 (0.0)	0 (0.0)	
Incontinentia pigmenti	0 (0.0)	0 (0.0)	
Infections	0 (0.0)	3 (4.2)	1[a]
Toxoplasmosis	0 (0.0)	3 (4.2)	1[a]
Coxackievirus	0 (0.0)	0 (0.0)	
Syphilis	0 (0.0)	0 (0.0)	
Varicella-Zoster	0 (0.0)	0 (0.0)	
HIV	0 (0.0)	0 (0.0)	
Parvo B19	0 (0.0)	0 (0.0)	
Rubella	0 (0.0)	0 (0.0)	
Cytomegalovirus	0 (0.0)	0 (0.0)	
Ophthalmic anomalies	3 (14.3)	10 (14.1)	1[a]
Congenital malformations of anterior segment	1 (4.8)	3 (4.2)	1[a]
Microphthalmia	1 (4.8)	4 (5.6)	1[a]
Persistent fetal vasculature	1 (4.8)	4 (5.6)	1[a]
Congenital malformation of retina	0 (0.0)	0 (0.0)	

IC = infantile cataract, HIV = Human Immunodeficiency Virus
[a] *P* values were calculated using Fisher's exact test.
